# Supplementary material for: Discovering biomarkers associated and predicting cardiovascular disease with high accuracy using a novel nexus of machine learning techniques for precision medicine
Source: Sci Rep. 2024 Jan 2;14:1. doi: 10.1038/s41598-023-50600-8 (PMC10762256; doi:10.1038/s41598-023-50600-8)
Supplement: Supplementary file 1 — Supplementary Information 1. [file 41598_2023_50600_MOESM1_ESM.pdf]

Supplementary Information

Separate Excel files are attached including:

Supplementary material 1: Dataset

Supplementary material 2: Biomarkers

Supplementary material 3: Classifier Metrics

Supplementary material 4: Gene-Disease-ICD
